# Type II (adult onset) Alexander disease in a paraplegic male with a rare D128N mutation in the GFAP gene 

**DOI:** 10.5414/NP300863

**Published:** 2015-05-22

**Authors:** Ki-Eun Chang, Drew Pratt, Bibhuti B. Mishra, Nancy Edwards, Mark Hallett, Abhik Ray-Chaudhury

**Affiliations:** 1 Laboratory of Cell Biology, Center for Cancer Research, National Cancer Institute, National Institutes of Health, Bethesda, MD,; 2 Laboratory of Pathology, National Cancer Institute, National Institutes of Health, Bethesda, MD,; 3 Neurology Section, Department of Neurosciences, Inova Fairfax Hospital, Falls Church, VA,; 4 Surgical Neurology Branch, National Institute of Neurological Disorders and Stroke, National Institutes of Health, Bethesda, MD, and; 5 Human Motor Control Section, National Institute of Neurological Disorders and Stroke, National Institutes of Health, Bethesda, MD, USA

**Keywords:** Alexander disease, glial fibrillary acidic protein, leukodystrophy, fibrinoid degeneration, Rosenthal fibers

## Abstract

Abstract not available.

*Authors have contributed equally to this work. 

## Letter to the Editor 

Sir, – In order to contribute to the genotype-phenotype correlation in this rare leukodystrophy variant, we report a case of type II Alexander disease (a subset of which was previously named “adult onset” or AOAD) in a man with a rare mutation in the GFAP gene (c.382 G>A 9p.Asp128Asn). Originally described in 1949 [1], Alexander disease (AxD) is a disorder of astroglia characterized histopathologically by the presence of Rosenthal fibers on hematoxylin and eosin staining, composed of aggregates of GFAP, αB-crystallin, and HSP27 [2]. The majority of affected individuals have mutations in GFAP [3]. The classification of AxD has recently undergone revision and is currently in transition (for examples [4, 5]). Later onset (type II) patients typically present with ataxia, dysphagia, dysphonia, and a relative lack of supratentorial involvement (i.e. cognitive dysfunction, seizures) [6]. Here we present the case of a relatively older patient with AxD and describe, to our knowledge, the first neuropathologic examination in the D128N genotype. 

A 52-year-old African American man presented with complaints of right hip pain and fever. Past medical history included COPD, chronic stage IV sacral ulcers, chronic osteomyelitis, and paraplegia initially thought to be secondary to spinal cord ischemia. Diagnostic imaging revealed right lower lobe and perihilar infiltrates, and end-stage avascular necrosis suggesting degenerative changes or infection. MRI revealed abnormal signal intensity and enlargement of the right sciatic nerve consistent with neuropathy. The fever and pulmonary infiltrates were attributed to a urinary tract infection and chronic aspiration pneumonitis, respectively. Prior serology for HTLV-1 was negative. He was later discharged in stable condition. A few years after his last medical follow-up, the patient was found unresponsive at home and subsequently expired, attributed to a probable cardiac arrhythmia. 

**Figure 1.**
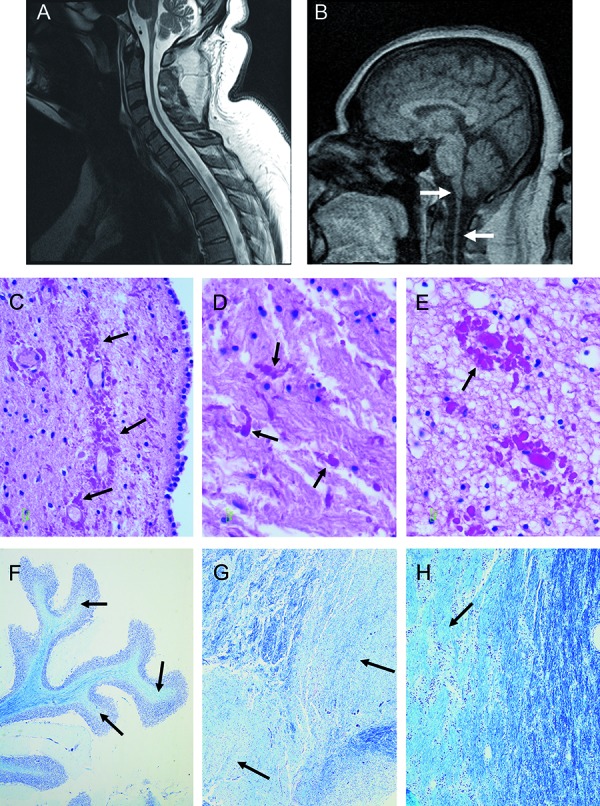
A: T2-weighted sagittal image of the lower brainstem, cervical and thoracic spinal cord. Diffuse atrophy of the cord is present from medulla to thoracic spinal cord. B: T1-weighted sagittal image of the brain and upper spinal cord taken without contrast. Severe atrophy of the medulla oblongata and cervical cord is noted by arrows. C: Scattered and perivascular Rosenthal fibers within the neuropil of cerebellum, hippocampus (D), (H&E, 600×) and the periventricular zone of basal ganglia (E) (H&E, 400×). F: Atrophic cerebellar folia with white matter demonstrating a lack of myelin (arrows). Intact myelination is shown in blue (Luxol Fast Blue stain, 200×). G: White matter demyelination in the pons/midbrain area of the brain stem (arrows) (Luxol Fast Blue stain, 400×). H: White matter demyelination of the corpus collosum (arrow) (Luxol Fast Blue stain, 100×).

Three years prior to his last hospitalization, the patient had undergone an MRI of the spine as part of the evaluation for his paraplegia. T1- and T2-weighted sequences with and without gadolinium-contrast revealed diffuse atrophy of the spinal cord and medulla with punctate areas of abnormal signal intensity, suggestive of a chronic diffuse demyelinating process (Figure 1A). The cervical discs and visualized osseous structures were normal. Facet hypertrophy was noted at the T10 – T22 levels with minimal deformity of the thecal sac on the left side. There were no enhancing lesions, disc herniation, or stenosis, and no previous scans were available for comparison. Due to progressive symptoms, a contrast-enhanced MRI of the brain was performed to rule out an intracranial demyelinating process. Once again, severe atrophy of the distal brainstem and cervical spinal cord was noted (Figure 1B); however, no focal intracranial abnormality was identified. A few years after his last medical follow-up, the patient was found unresponsive at home and subsequently expired despite resuscitation efforts. The cause of death was attributed to a probable cardiac arrhythmia. 

**Figure 2.**
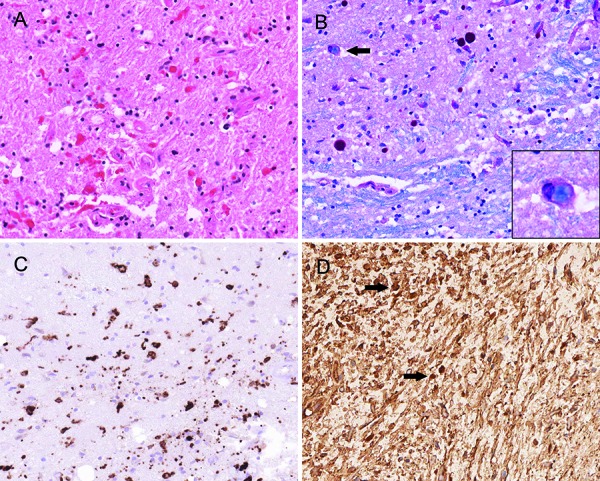
A: Section of medulla demonstrating scattered Rosenthal fibers (H&E, 200×) B: Luxol fast blue with PAS highlights an active demyelinating process with engulfment of myelin by histiocytes (arrow, inset; 200×). C: Areas corresponding to demyelination demonstrate a dense infiltrate of CD163+ macrophages (200×). D: While a relative preservation of axons is seen, numerous axonal swellings (arrows) indicate damage to the axons (NFP, neurofilament protein, 200×).

Postmortem examination of the cerebrum, cerebellum, and brainstem showed mild-to-moderate atrophy with foci of grayish discolorations noted in the white matter. Histopathologic examination revealed accumulation of Rosenthal fibers, in a scattered and perivascular arrangement in regions adjacent to the lateral ventricle (Figure 1C), cerebellum, and left hippocampus. Background reactive gliosis as well as white matter rarefaction (Figure 1D, Figure 1E) was present. Depopulation of neurons was noted in the dentate nucleus of the cerebellum. The pons was remarkable for periventricular and scattered Rosenthal fibers. Foci of demyelination in the parietal lobe, cerebellar white matters, and corpus callosum were confirmed with Luxol fast blue staining (Figure 1F, Figure 1G, Figure 1H). Examination of the medulla, including the tegmentum, demonstrated large numbers of Rosenthal fibers and demyelination (Figure 2A, Figure 2B); foamy macrophages with intracellular LFB-positive myelin were also identified (Figure 2C). Relative preservation of axons were observed with a neurofilament protein immunostain (Figure 2D). Immunostains for ubiquitin, α-synuclein and τ-proteins showed no glial intracytoplasmic inclusions typically seen in disorders such as multiple system atrophy (MSA) or immunopositivity for neuronal τ-protein or ballooned neurons in the neocortices as seen in corticobasal ganglionic degeneration (CBD). Molecular testing revealed a missense mutation in the GFAP gene involving nucleotide position 383 of codon 128, resulting in an amino acid change from aspartic acid (D) to asparagine (N)(i.e., GFAP c.382 G>A 9p.Asp128Asn) (dbSNP Reference: rs267607509). In silico analysis using polymorphism phenotyping tool (PolyPhen-2.2.2) revealed that this sequence alteration is “probably damaging”. To date, this specific mutation has only been noted in two other cases of AxD [6, 7]; however, these two cases most likely represent the same patient, as both are presented by the same authors/institution and highlight patients with the same age and disease duration. 

AxD is a rare group of degenerative leukodystrophies that has traditionally been categorized into three distinct diagnoses based on age at symptom onset: infantile (birth to 2 years), juvenile (2 – 14 years), and adult (> 14 years) [4]. Infantile AxD is fatal, characterized by macrocephaly, psychomotor regression, spasticity, ataxia and seizures [4]. Conversely, adult onset AxD is often protracted, presenting with vague and progressive muscular and bulbar symptoms. Severe pyramidal involvement in type II AxD can result in paresis and dysarthria suggestive of an ischemic neurologic event, as seen in this case, while bulbar involvement may contribute to aspiration pneumonia presenting with fever and respiratory failure [8]. The clinical differential diagnosis for our patient included, but was not limited to, hereditary spastic paraplegia and spinocerebellar ataxia. MRI findings are helpful in this regard, as patients with type II disease commonly have atrophy limited to the brainstem and upper cervical spinal cord that worsen with disease duration [7]. Sequence abnormalities may range from subtle T2 high-signal-intensity changes in the upper corticospinal tract to extensive confluent white matter changes in a frontoparietal distribution, along the corticospinal tract, as well as in the medulla and upper and middle cerebellar peduncles. 

Mutations in the GFAP gene are thought to account for greater than 95% of cases of AxD [9]. The downstream effect of this insult is thought to include decreased astrocyte stress response [2], proteasome dysfunction [5], GFAP aggregation [10], and possible loss of astrocyte myelination signaling to oligodendroglia [11]. It has been postulated that mutation site location in the GFAP gene may influence the discrepancy in distribution of injury seen between AxD subtypes [6]. The D128N mutation in our patient is an exceedingly rare finding. A case series of late onset AxD revealed the D128N mutation in a 64-year-old male with lower limb weakness, gait abnormalities, asymmetric motor impairment, spasticity, globally increased deep tendon reflexes, and Babinski sign [6]. MR changes included marked signal changes/atrophy of the bulbar region and cervical spinal cord. While nonspecific, the severe lower limb involvement seen in our patient, complicated in our case by neuropathy and paraplegia, is in keeping with the initial presentation of the previously reported patient with the D128N genotype. Additionally, the neuropathologic findings in our patient, including atrophy of infratentorial structures on gross examination and on imaging, are consistent with reports on type II AxD [6, 8]. No signal abnormalities were detected in the periventricular white matter in our patient, a finding also seen in previous report(s) of older patients with type II disease and this feature, in particular, is not typically seen in individuals over the age of 40 [6]. 

Rosenthal fibers, while characteristic of Alexander disease, are nonspecific and can be seen in other disorders such as multiple sclerosis and pilocytic astrocytomas, among others [12]. Although not frequent, palatal myoclonus is thought to represent a highly specific finding in type II AxD [6]. Other, more common symptoms include dysarthria, dysphonia, vocal cord palsy, and dysphagia. Cerebellar involvement frequently produces ataxia and ocular abnormalities such as nystagmus. In contrast, type I (early onset) patients present with a predominantly frontal leukodystrophy manifesting as seizures and developmental delay [9]. Early onset patients are diagnosed when neuroradiologic changes exist in the white matter of the frontal regions, periventricular rimming and/or contrast enhancement, basal ganglia and thalamic involvement, and scattered abnormalities of the brainstem [11]; this difference in the distribution of white matter changes has likely been the impetus for reclassifying the disease partly based on the areas affected. Consistent with the type II phenotype, our patient had an extensive medical history attributable to bulbar and pyramidal pathology such as paraplegia, chronic aspiration pneumonitis, and recurrent urinary tract infections. Final confirmation of the AxD can be made on post-mortem histopathology with the identification of Rosenthal fibers in the subpial and white matter regions as well as loss of myelin [5, 11]. 

Proposed revisions to the classification of AxD include categories that exploit the supratentorial features of the early onset subtype and the bulbospinal features of late onset disease [5]. Reports of patients with AxD will continue to increase in the era of molecular testing and results will lend support to diagnostic schema based on phenotype rather than age. 

## Acknowledgment 

Medical Research Scholars Program (MRSP) Acknowledgement Statement (sponsoring author KC): This program was made possible through the National Institutes of Health (NIH) Medical Research Scholars Program, a public-private partnership supported jointly by the NIH and generous contributions to the Foundation for the NIH from Pfizer Inc., The Doris Duke Charitable Foundation, The Alexandria Real Estate Equities, Inc. and Mr. and Mrs. Joel S. Marcus, and the Howard Hughes Medical Institute, as well as other private donors. 

## Conflict of interest 

The authors report no competing interest for any portion of this work.
